# Homotypic cell-in-cell structures as an adverse prognostic predictor of hepatocellular carcinoma

**DOI:** 10.3389/fonc.2022.1007305

**Published:** 2022-11-07

**Authors:** Ruizhi Wang, Yichao Zhu, Hao Zhong, Xinyue Gao, Qiang Sun, Meifang He

**Affiliations:** ^1^ Laboratory of General Surgery, The First Affiliated Hospital of Sun Yat-Sen University, Guangzhou, China; ^2^ Department of Laboratory Medicine, The First Affiliated Hospital of Sun Yat-Sen University, Guangzhou, China; ^3^ Beijing Institute of Biotechnology; Research Unit of Cell Death Mechanism, Chinese Academy of Medical Science, Beijing, China

**Keywords:** homotypic cell-in-cell structures (hoCICs), E-cadherin, prognostic predictor, overall survival (OS), hepatocellular carcinoma (HCC)

## Abstract

Hepatocellular carcinoma (HCC) is one of the most common malignant liver tumors. A homotypic cell-in-cell structure (hoCIC) refers to one or more cells internalized into the same type as their neighbors, which predominantly occurs in multiple tumors. The objective of this study was to investigate the prognostic value of hoCICs in HCC and its relationship with other clinicopathological features. By immunostaining analysis of a panel of HCC tissues, we found that hoCICs were prevalent in tumor tissues (54/90) but not in para-tumor tissues (17/90). The presence of hoCICs in tumor tissues was closely associated with E-cadherin expression. The presence of CICs was identified as significantly associated with poor survival rates of patients with HCC, comparable to traditional clinicopathological parameters, such as histological grade [hazard ratio (HR) = 0.734, *p* = 0.320]. Multivariate Cox regression analysis further confirmed that CICs were an independent risk factor for poor survival (HR = 1.902, *p* = 0.047). In addition, hoCICs were the predominant contributor in a nomogram model constructed for survival prediction at 1, 3, and 5 years [the areas under the curve (AUCs) were 0.760, 0.733, and 0.794, respectively]. Stratification analysis indicated that hoCICs tend to selectively affect patients with high-grade disease (HR = 2.477, *p =* 0.009) and at the early TNM stage (HR = 2.351, *p =* 0.05). Thus, hoCICs predict poor survival of patients with HCC, particularly those with higher grades and at an early stage.

## Introduction

Globally, hepatocellular carcinoma (HCC) is one of the most common malignant tumors in humans. Although rapid advancements in HCC prognostic predictors have been reported, the prognosis for patients with HCC remains poor, with a 5-year survival rate of less than 30% (1). Therefore, there is an urgent needed to explore more sensitive biomarkers, especially pathological or morphological markers that could directly and functionally assess the malignancy of HCC.

Cell-in-cell structures (CICs) are cellular structures in which one or more viable cells are present in another cell (2–4), which might serve as an independent prognostic factor affecting patient outcomes in many human tumors, such as pancreatic ductal adenocarcinoma (5), breast cancer (6), esophageal squamous cell carcinoma (7), buccal mucosa squamous cell carcinoma (8), head and neck squamous cell carcinoma (9), and colorectal adenocarcinoma (10). Based on a set of core elements (11, 12, 31), CICs could be formed homotypically (between the same type of cells) or heterotypically (between different types of cells) (13), both of which generally lead to the death of the internalized cells in an acidified lysosome (14–16). This suggests that CICs might function *via* a mechanism of cell competition to promote tumor progression (17–19). Tumor cells are believed to take advantage of CICs to accumulate aneuploidy and select fitter clones for tumor evolution (20–22), to feed themselves under stress conditions (23, 24), to evade immune attacks *via* eliminating cytotoxic cells (15, 25, 26), or to potentiate immune killing under certain circumstances (27, 28). Therefore, CICs are a potential candidate to functionally determine malignant progression and patient survival. However, whether CICs could serve as a prognostic factor for patients with HCC remains unclear.

In this study, we aimed to explore the feasibility of using homotypic CICs (hoCICs) as a functional biomarker that could read out tumor malignancy and predict patient survival in HCC. Ninety pairs of paired tumor and para-tumor specimens from patients with HCC were employed and stained with E-cadherin to identify epithelial tumor cells. Our work revealed that hoCICs are linked to E-cadherin expression in HCC tissues. Moreover, we determined the role of hoCICs as a selective prognostic classifier that predicts poor overall survival (OS) for patients with HCC, especially for those with higher grades and at an early stage.

## Materials and methods

### Human tumor tissue microarray

A human tumor tissue microarray (TMA) with paired samples of tumor and para-tumor tissues for 90 patients with HCC was purchased from Shanghai Outdo Biotech Co. Ltd. (Shanghai, China). The TMA slide was prepared from formalin-fixed, paraffin-embedded tumor and paired para-tumor tissues. There were 180 cores on one slide, which included 90 cases of HCC tumor tissues and 90 cases of para-tumor tissues. The tumors were graded pathologically according to the World Health Organization classification system, and the pathological staging was performed based on the American Joint Committee on Cancer (AJCC) TNM classification criteria (7th Edition).

### TMA staining and antibodies

The TMA slide was routinely de-paraffinized using the xylene-ethanol method following baking in 65°C for 2 h. Antigen retrieval was performed in citrate acid buffer by microwaving for 15 min after boiling, followed by 1 h of blocking in 5% bovine serum albumin in Tris-buffered saline. The slide was first stained with antibodies against CD45 (Boster, Wuhan, China; BM0091) using an Opal Multiplex tissue staining kit (Perkin Elmer, Waltham, MA, USA; NEL791001KT) according to the manufacturer’s instructions, and CD45 molecules were labeled with the Cyanine 5 fluorophore. Then, the slide was incubated with mixed antibodies against E-cadherin (BD Biosciences, San Jose, CA, USA; 610181) and CD44 (Abcam, Cambridge, MA, USA; 243894), followed by secondary antibodies comprising Alexa Fluor 488 anti-mouse antibodies (ThermoFisher, Waltham, MA, USA; A31561) and Alexa Fluor 564 anti-rabbit antibody (ThermoFisher, A11035). Samples were labeled with 4′,6-diamidino-2-phenylindole (DAPI) to show nuclei, followed by mounting with Antifade reagent (ThermoFisher, P36934), adding cover slips, and sealing with nail varnish. Images were acquired using the TMA modules of Vectra^®^ Automated Imaging System (Perkin Elmer) with a 20× objective lens.

### Quantification of CICs

CICs were quantified as described previously (5). CICs were scored as one or more cells inside of another cell with a crescent nucleus. The cell boundary was indicated by E-cadherin, which labels the cell membrane, and DAPI, which labels cell nucleus. For efficient quantification in the TMA, CICs were usually counted in a composite image of mixed fluorescent channels and then confirmed in the unmixed channel. All fields of every core were screened and counted to obtain the number of CICs.

### Statistical analysis

Statistical analysis was performed using SPSS Statistics software (version 19.0, SPSS Inc., Chicago, IL, USA). OS was defined as the time from the date of surgery to death or the most recent contact or visit. Survival curves were analyzed using the Kaplan–Meier method, and the differences in survival curves were compared using the log-rank test. Univariate and multivariate survival analyses were performed using a Cox proportional hazards model. The association between clinicopathological factors and the number of CICs was analyzed using the Chi-squared test. AUC calculation was performed and graphed with R software. For all these analyses, *p* < 0.05 was considered statistically significant.

## Results

### Homotypic CICs are associated with clinicopathological characteristics in HCC tissues

**Figure 1:**
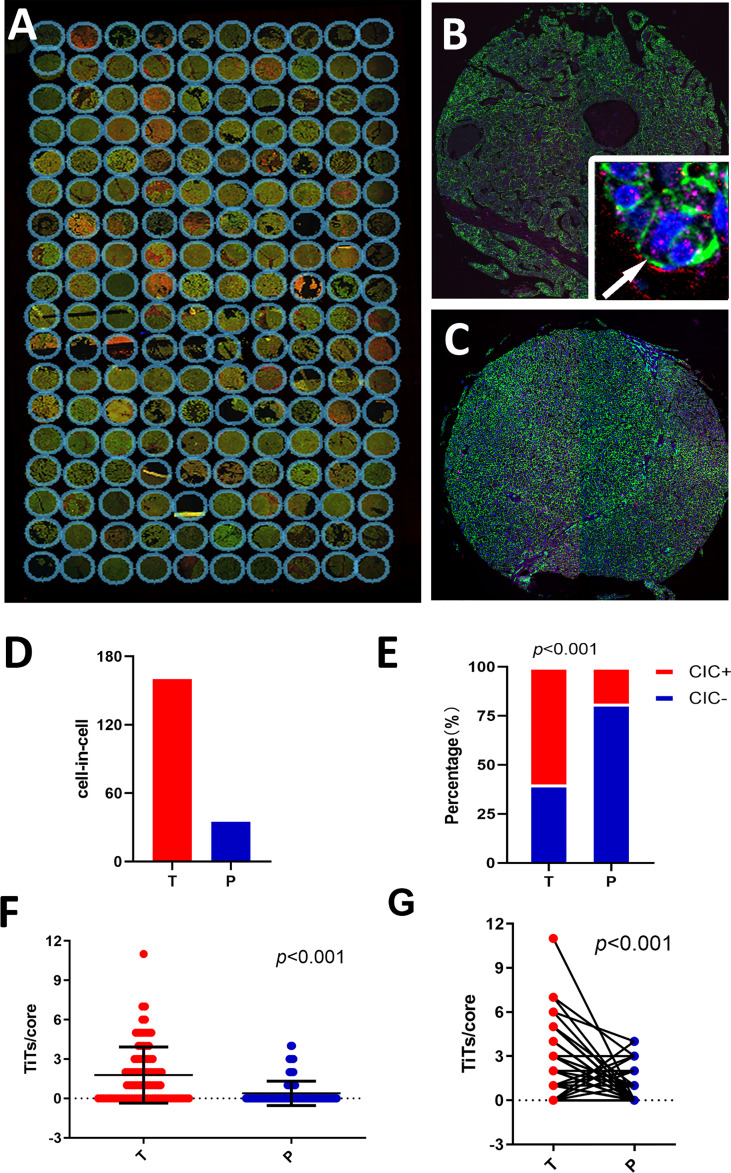
Detecting homotypic CICs in a tissue microarray (TMA) of human HCC. **(A)** Composite images of a whole TMA slide stained with antibodies against E-cadherin (green), CD44 (red), CD45 (pink), and DAPI staining of the nucleus (blue). **(B)** Single representative tumor tissue (T) core. The arrow in the inserted image shows one typical CIC. **(C)** Para-tumor tissue core (P). **(D)** CIC counts in tumor and para-tumor tissues. **(E)** Percentages of patients with or without CICs in tumor and non-tumor tissues. **(F)** CICs detected in 90 tumor cores and paired para-tumor tissue cores. **(G)** CICs presented as number of CICs per core in the tumor and non-tumor tissues.

**Figure 2:**
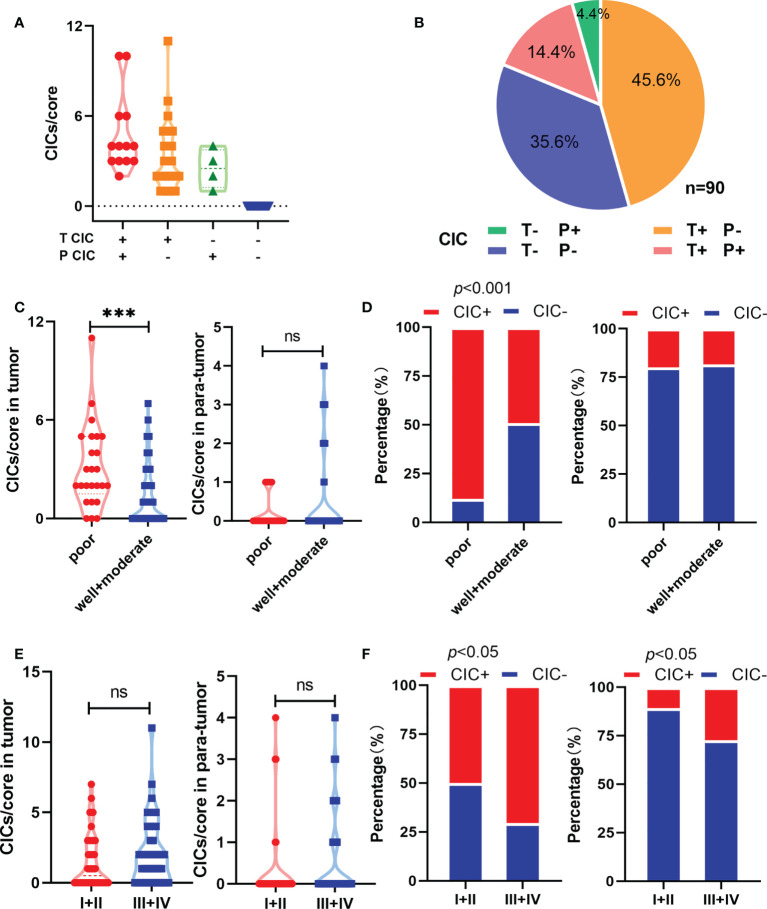
Homotypic CICs are associated with clinicopathological characteristics in HCC tissues. **(A)** CIC counts normalized by core area in CIC positive tumor and para-tumor tissues. **(B)** Quantification of CICs in tumor and para-tumor tissues. **(C)** Quantification of CICs stratified by histological grade in tumor (left) and para-tumor tissues (right). **(D)** CIC compositions by histological grade in tumor (left) and para-tumor tissues (right). **(E)** Quantification of CICs stratified by TNM in tumor (left) and para-tumor tissues (right). **(F)** CIC compositions by TNM in tumor (left) and para-tumor tissues (right). *P < 0.05; **P < 0.01; ***P < 0.001; ns, not significant.

In total, 180 specimens from 90 pair-matched tumor (T) and para-tumor (P) tissues plotted on a human tumor tissue microarray (TMA) were included in this study (**Figures 1A–C**). The clinical characteristics of the 90 patients are listed in **Table 1**. Most of the patients were male (90%), and their median age was 54 (25–73) years old. All of the patients had follow-ups until their death or until their most recent contact or visit. At the time of their most recent contact, 57 of the 90 patients had died from HCC. The median follow-up time was 29 months (1–80 months). Homotypic CICs in each core of the specimens were quantified as described previously (29), and only structures with inner cells fully inside outer cells were counted (**Figure 1B**). Tumor tissues contained many more CICs than para-tumor tissues (**Figure 1D**), and more tumor tissues (54/90) contained CICs than did the para-tumor tissues (17/90) (*p* < 0.001) (**Figure 1E**). The mean number of CICs in tumor tissues (1.78 ± 0.23) was much higher than that in para-tumor tissues (0.39 ± 0.098) (*p* < 0.001) (**Figures 1F, G**). The majority of patients had CICs in their tumor tissues but not in their para-tumor tissues (**Figures 2A, B**). CICs were easier to detect in tumor tissues of patients with advanced stage disease (*p* = 0.048) and the presence of CICs demonstrated a significant association with a low level of tumor differentiation (*p =* 0.001) (**Table 1**). Meanwhile, CICs identified in poorly differentiated tumor cells were much more prevalent than those in moderate/well-differentiated tumor cells in HCC tissues, which was not the case in para-tumor tissues (**Figures 2C, D**). Rather, whether in tumor or para-tumor tissues, CICs were more common in patients with advanced stage in HCC (**Figures 2E, F**). These results are consistent with the notion that tumor cells at advanced stages have higher cannibal activities (23).

**Table 1:** Correlations between CICs and clinicopathological features in patients with hepatocellular carcinoma (HCC).

Clinicopathological feature	Total (*n* = 90)	Cell-in-cell (CICs)		*p*-value(*χ* ^2^ text)
		Absent (*n* = 36, 40%) Present (*n* = 54, 60%)		
**Age (years)**				
≤54	47	18 (38.3)	29 (61.7)	0.730
>54	43	18 (41.9)	25 (58.1)
**Sex**				
Male	81	32 (39.5)	49 (60.5)	0.774
Female	9	4 (44.4)	5 (55.6)
**Size (cm)**				
≤5	34	18 (52.9)	16 (47.1)	0.051
>5	56	18 (32.1)	38 (67.9)
**Clinical stage (pTNM)**				
I+II	46	23 (50.0)	23 (50.0)	**0.048** *
III	44	13 (29.5)	31 (70.5)
**Histological grade**				
Well + Moderate	65	33 (50.8)	32 (49.2)	**0.001** ***
Poor	25	3 (12.0)	22 (88.0)
**T classification**				
T1 + T2	46	23 (50.0)	23 (50.0)	**0.048** *
T3 + T4	44	13 (29.5)	31 (70.5)
**N classification**				
Absent	88	36 (40.9)	52 (59.1)	0.243
Present	2	0 (0)	2 (100)
**Metastasis**				
Absent	88	36 (40.9)	52 (59.1)	0.243
Present	2	1 (5.3)	18 (94.7)

*Statistically significant; pTNM: tumor-node-metastasis classification pancreatic cancer staging. *P < 0.05; **P < 0.01; ***P < 0.001. Red values indicate significant differences between comparisons with less than 0.05.

### Homotypic CIC formation is closely associated with E-cadherin expression in HCC tissues

**Figure 3:**
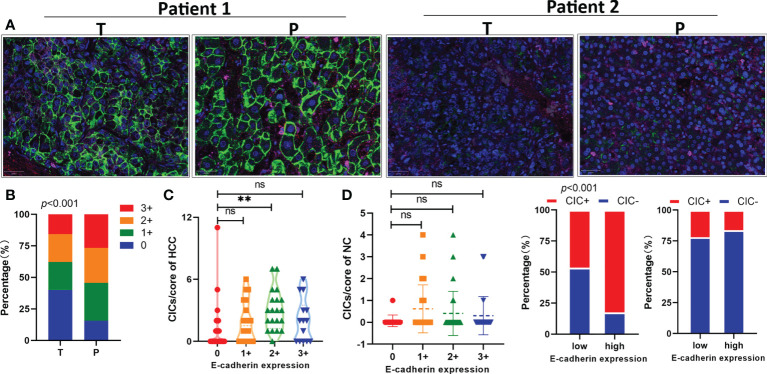
E-cadherin expression in tumor tissues is positively associated with homotypic CIC formation. **(A)** Representative images for high (two on the left) or low (two on the right) levels of E-cadherin expression in pair-matched tumor (T) and para-tumor (P) tissue samples of the TMA by immunofluorescent staining. The TMA slides were stained with antibodies against E-cadherin (green), CD44 (red), CD45 (pink), and DAPI staining of the nucleus (blue). **(B)** Quantification of E-cadherin expression in pair-matched tumor (T) and para-tumor (P) tissue samples of the TMA. *n* = 180 with 90 for tumor and 90 for para-tumor, respectively. **(C)** E-cadherin expression correlated significantly with CIC formation in tumor tissues (left), but not in para-tumor tissues of the TMA. **(D)** CIC compositions in low or high E-cadherin expression groups of tumor (left) and para-tumor tissues (right). *P < 0.05; **P < 0.01; ***P < 0.001; ns, not significant.

E-cadherin has been reported to be a key adhesion molecule mediating the formation of homotypic CICs (30); therefore, we performed immunostaining on the TMA to analyze the link between E-cadherin and hoCIC formation (**Figure 3A**). Consistent with the result from a previous report (31), the level of E-cadherin was also reduced in HCC tissues compared with that in the para-tumor tissues (**Figure 3B**). Moreover, the level of E-cadherin was closely related to hoCIC formation in tumor tissues (**Figure 3C**). Further analysis showed that more CICs were identified in HCC tissues with high levels of E-cadherin, while there was no difference in the composition in the para-tumor tissues (**Figure 3D**). These results indicated that homotypic CICs are closely associated with E-cadherin levels in HCC tissues.

### The presence of homotypic CICs is a strong predictor of shorter postoperative survival

**Figure 4:**
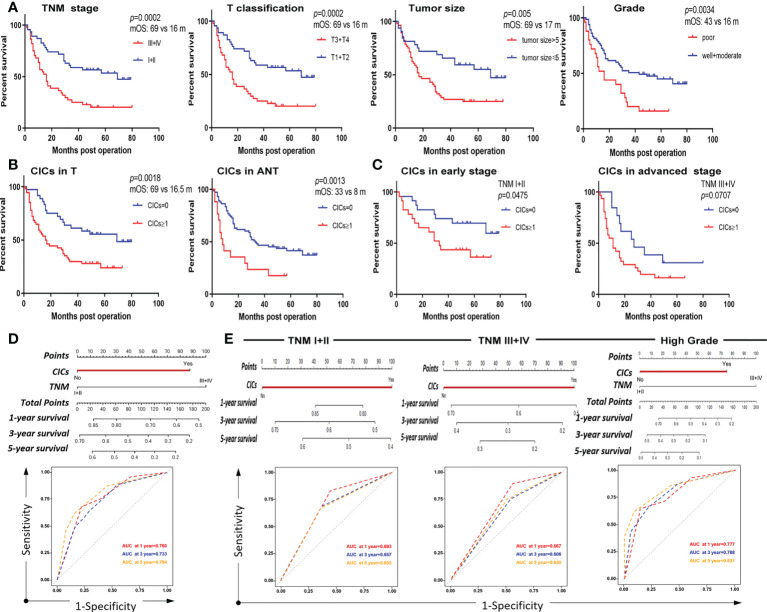
CICs selectively impact overall survival of a certain group of patients with HCC. **(A)** Survival analysis of patients with HCC stratified by TNM, T classification, tumor size, and histological grade, respectively. **(B)** The presence of CIC formation (CICs ≥ 1/core) both in tumor (left) and para-tumor (right) tissues is associated with shorter patient survival by Kaplan–Meier plotting of data from TMA staining. **(C)** The presence of CIC formation (CICs ≥ 1/core) in tumor (left) but not in para-tumor (right) tissues is associated with shorter patient survival by Kaplan–Meier plotting of data from TMA staining. **(D)** Nomogram and AUC analysis with two independent prognostic factors (TNM stage and CICs). **(E)** Nomogram and AUC analysis in a cohort of patients stratified by TNM stage (I+II vs. III+IV) (left and middle) or histological grade (well + moderate) (right).

Univariate analysis revealed that some traditional variables, including TNM stage, grade, tumor size, and T classification, were significantly associated with shorter postoperative survival (**Table 2**). Among them, the TNM stage and T classification were the most consistent and representative survival classifiers [median overall survival time (mOS): 69 vs. 16 months; *p =* 0.0002] in the cohorts (**Table 2** and **Figure 4A**). Remarkably, the presence of hoCICs was a strong prognostic factor to predict shorter postoperative survival (mOS: 69 vs. 16.5 months; *p =* 0.0018), which displayed a performance that was comparable to, or even stronger than, those for the classifiers of tumor size (mOS: 69 vs. 17 months; *p =* 0.005) or histological grade (mOS: 43 vs. 16 months; *p =* 0.0034) (**Figures 4A, B**). Notably, hoCICs consistently displayed a strong prognostic power in predicting shorter postoperative survival across the cohorts of both tumor (mOS: 69 vs. 16.5 months; *p =* 0.0018) and para-tumor (mOS: 33 vs. 8 months; *p =* 0.0013) (**Figure 4B**). As shown in **Figure 4C**, hoCICs seemed to preferentially predict shorter survival of patients with HCC at the early TNM (I+II) stage (*p =* 0.0475), but not those at the late stage of TNM (III+IV) (*p =* 0.0707).

**Table 2:** Univariate analysis of survival in patients with hepatocellular carcinoma (HCC) by Kaplan–Meier survival analysis and Cox regression analysis.

Characteristics	*n*	Median survival (months; 95% CI)	*p*(KP)	HR(%% CI)	*p*(Cox)
**Age**					
≤54	47	25.000 (12.708–37.292)	0.503	0.838 (0.497–1.414)	0.508
>54	43	34.000 (19.009–48.991)			
**Sex**					
Male	81	29.000 (15.281–42.719)	0.625	0.798(0.318–1.999)	0.630
Female	9	29.000 (17.313–40.687)			
**Size**					
≤5	34	69.000 (43.457–73.534)	**0.002**	2.455(1.350–4.463)	**0.003**
>5	56	17.000 (7.839–26.161)			
**Histological grade**					
Well + Moderate	65	43.000 (10.169–75.831)	**0.003**	0.455 (0.264–0.786)	**0.005**
Poor	25	16.000 (7.892–24.108)			
**T classification**					
T1 + T2	46	69.000 (61.569–78.491)	**0.000**	2.641(1.532–4.555)	**0.000**
T3 + T4	44	16.000 (10.451–21.549)			
**N classification**					
Absent	88	29.000 (20.726–37.274)	0.249	2.236(0.538-9.288)	0.268
Present	2	13.000 (10.120–21.880)			
**Metastasis**					
Absent	88	29.000 (20.726–37.274)	0.065	3.459(0.833–	0.088
Present	2	2.000 (0.000–8.500)		14.374)	
**TNM stage**					
I + II	46	69.000 (61.569–78.491)	**0.000**	2.641(1.532–4.555)	**0.000**
III	44	16.000 (10.451–21.549)			
**CICs**					
Present	54	69.000 (55.656–80.068)	**0.002**	2.404(1.355–4.265)	**0.003**
Absent	36	16.000 (8.799–23.201)			

Red values indicate significant differences between comparisons with less than 0.05.

### Homotypic CICs are a prominent independent prognostic factor for HCC

To analyze whether hoCICs are an independent prognostic factor for postoperative survival, we included the variables identified in the univariate analysis (CICs, grade, and TNM stage) in multivariate survival analysis using the Cox proportional hazards model. As expected, the TNM stage was identified as an independent prognostic factor [hazard ratio (HR) = 2.275, 95% confidence interval (CI): 1.299–3.983, *p =* 0.004]. Notably, the hoCICs were also identified as an independent prognostic factor, with an HR of 1.902 (95% CI: 1.009–3.585, *p =* 0.047) (**Table 3**).

**Table 3:** Multivariate analysis of prognostic parameters for HCC.

Prognostic parameter	HR (95% CI)	*p*-value
**CICs** (present vs. absent)	1.902 (1.009–3.585)	0.047*
**Histological grade** (well + moderate vs. poor)	0.734 (0.400–1.349)	0.32
**TNM stage** (I + II vs. III + IV)	2. 275 (1.299–3.983)	0.004**

*P < 0.05; **P < 0.01; ***P < 0.001. Red values indicate significant differences between comparisons with less than 0.05.

Furthermore, we constructed a nomogram that incorporated the two identified independent prognostic factors (CICs and TNM) to directly evaluate the prognostic contribution of hoCICs. In the nomogram, each variable was assigned a score on a point scale based on its prediction power extracted from the multivariate analysis. As shown in **Figure 4D**, CICs were a dominant factor in predicting patient survival. Location of the total score from all variables on the total point scale estimated the probability of patient survival at 1, 3, and 5 years, respectively, which showed that CICs indeed improved the prediction performance [areas under the curve (AUCs) of 0.760, 0.733, and 0.794 for 1-, 3-, and 5-year survival, respectively].

### Homotypic CICs preferentially impact the survival of patients with lower grades and at an early HCC

HoCICs are closely associated with TNM stage and histological grade (**Table 1**); therefore, we further stratified the patient cohort by TNM stage (I + II vs. III + IV) and histological grade (well + moderate vs. poor) for multivariate survival analysis using a Cox proportional hazards model. As shown in **Table 4**, using hoCICs as a prognostic factor independently predicted postoperative OS specifically in patients with early TNM stage (I + II) disease and the HR for death was increased (HR = 2.351, 95% CI: 0.978–5.648; *p =* 0.05) compared with that of the unstratified cohort (HR = 2.275, 95% CI: 1.299–3.985; *p =* 0.004) (**Table 3**). Similarly, the selectivity of CICs was also applied to grade-stratified patients of high (well + moderate) histological grades (HR = 2.477, 95% CI: 1.260–4.870; *p =* 0.009) (**Table 5**). Nomogram construction and AUC analysis confirmed that CICs are a selective prognostic classifier that predicts a poor survival outcome for patients with HCC with high grade and at an early stage (**Figure 4E**).

**Table 4:** Multivariate analysis of prognostic parameters for HCC stratified by TNM.

Prognostic parameter	TNM I/II (*n* = 46)		TNM III/IV (*n* = 44)	
	HR (95% CI)	*p*	HR (95% CI)	*p*
**CICs** (present vs. absent)	2.351 (0.978–5.648)	**0.050**	1.970 (0.919–4.224)	**0.081**

Red values indicate significant differences between comparisons with less than 0.05. Bold values indicate significant differences between comparisons with less than 0.05.

**Table 5:** Multivariate analysis of prognostic parameters for HCC stratified by histological grade.

Prognostic parameter	Well + Moderate (*n* = 46)		Poor (*n* = 25)	
	HR (95% CI)	*p*	HR (95% CI)	*p*
**CICs** (present vs. absent)	2.477 (1.260–4.870)	**0.009**	0.667 (0. 190–2.334)	**0.526**

Red values indicate significant differences between comparisons with less than 0.05. Bold values indicate significant differences between comparisons with less than 0.05.

## Discussion

In recent years, HCC has become one of the most frequently occurring types of cancer and its prevalence is increasing. If patients with HCC are diagnosed at an early stage, they can be managed curatively with surgical resection or liver transplantation. However, because of advanced stage HCC and underlying liver dysfunction, only 15% of patients are eligible for curative surgery (32). There is an urgent need to explore new diagnostic and therapeutic factors for HCC. Our data supported the view that hoCICs are promising prognostic markers for HCC, and the presence of hoCICs is generally associated with E-cadherin expression in tumor tissues. The presence of hoCICs tends to predict poor survival outcomes, which is also in agreement with the notion that hoCIC, *via* entosis, is a mechanism of cell competition to promote clonal selection toward malignancy (17, 19, 33).

In this study, downregulation of E-cadherin levels was common in HCC tissues compared with that in para-tumor tissues (**Figure 3B**), which was consistent with a previous report (31). E-cadherin expression is still an essential element controlling the formation of hoCICs, in which E-cadherin is recruited to cell–cell junctions during cell internalization (30). Consistent with this view, our data showed here that the presence of hoCICs was positively associated with E-cadherin levels in tumor tissues.

One of the most important implications of this work is the application of hoCICs as a functional index for patient prognosis. Currently, traditional or molecular pathology is the main method for histological diagnosis to produce information at the level of individual cells or molecules. However, both the morphology and genetics of tumors are generally heterogeneous; therefore, new histopathological and immunohistochemical parameters for the precise prediction of tumor malignancy and patient prognosis are urgently required. CICs might be a favorable parameter because of their simple functionality in assessing tumor malignancy. CIC formation is a specific type of cell–cell interaction leading to different functional outcomes of inner and outer cells. On the one hand, cell death is the main fate of inner cells, which could provide nutrition to confer growth advantages to the outer survivors after nutrient deprivation (34). Some tumor cells utilize CICs as an important mechanism to maintain their proliferation under stressed conditions; therefore, it is conceivable that CICs in tumor cells promote cell competition for clonal selection and tumor evolution (17, 33). The winner tumor cell clones repetitively engulf the loser cell clones, mediated by the CIC process, leading to uncontrolled tumor growth. Our data support this view, in that the presence of hoCICs predicts shorter patient OS in HCC. On the other hand, inner cells block the mitosis of outer cells by disrupting cytokinesis, resulting in aneuploidy (22), which also agrees with the concept that aneuploidy is an effective method for tumor malignancy. CIC-mediated aneuploidy provides more opportunity for the winner cell clones to acquire new mutations and malignant phenotypes, in which the tumors continue to grow and progress. In agreement with this, the presence of CICs was significantly associated with histological grade and TNM stage.

In conclusion, the present study reported that CICs are a potential adverse prognostic marker to predict the survival of patients with HCC, particularly those with high histological grade and at an early stage. Our work also supports the functional pathology of CICs as a valuable biological characteristic supplementing traditional pathology.

## Data availability statement

The raw data supporting the conclusions of this article will be made available by the authors, without undue reservation.

## Ethics statement

The studies involving human participants were reviewed and approved by the Institutional Ethical Review Boards of the First Affiliated Hospital of Sun Yat-sen University. The patients/participants provided their written informed consent to participate in this study.

## Author contributions

Concept and design: MH. Carried out the experiments: RW and HZ. Data acquisition and analysis: YZ and XG. Data interpretation and drafting of the manuscript: QS and MH. Funding: MH, RW and QS. All authors have read and approved the final manuscript.

## Funding

This work was supported by grants from the National Natural Science Foundation of China (grant numbers 81972483, 81972750, and 31970685), the National Key R&D Program of China (grant number 2022YFC3600100), the Natural Science Foundation of Guangdong Province (grant number 2021A1515010127), and the Guangzhou Municipal Science and Technology Project (grant number 202102020028).

## Conflict of interest

The authors declare that the research was conducted in the absence of any commercial or financial relationships that could be construed as a potential conflict of interest.

## Publisher’s note

All claims expressed in this article are solely those of the authors and do not necessarily represent those of their affiliated organizations, or those of the publisher, the editors and the reviewers. Any product that may be evaluated in this article, or claim that may be made by its manufacturer, is not guaranteed or endorsed by the publisher.
